# Combinatory Effects of Bone Marrow-Derived Mesenchymal Stem Cells and Indomethacin on Adjuvant-Induced Arthritis in Wistar Rats: Roles of IL-1*β*, IL-4, Nrf-2, and Oxidative Stress

**DOI:** 10.1155/2021/8899143

**Published:** 2021-01-05

**Authors:** Eman A. Ahmed, Osama M. Ahmed, Hanaa I. Fahim, Emad A. Mahdi, Tarek M. Ali, Basem H. Elesawy, Mohamed B. Ashour

**Affiliations:** ^1^Physiology Division, Zoology Department, Faculty of Science, Beni-Suef University, P.O. Box 62521, Beni-Suef, Egypt; ^2^Pathology Department, Faculty of Veterinary Medicine, Beni-Suef University, Beni-Suef, Egypt; ^3^Department of Physiology, College of Medicine, Taif University, P.O. Box 11099, Taif 21944, Saudi Arabia; ^4^Department of Physiology, Faculty of Medicine, Beni-Suef University, Beni-Suef, Egypt; ^5^Department of Clinical Laboratory Sciences, College of Applied Medical Sciences, Taif University, P.O. Box 11099, Taif 21944, Saudi Arabia; ^6^Department of Pathology, Faculty of Medicine, Mansoura University, Mansoura, Egypt

## Abstract

Rheumatoid arthritis (RA) is a disorder triggered by autoimmune reactions and related with chronic inflammation and severe disability. Bone Marrow-derived Mesenchymal Stem Cells (BM-MSCs) have shown a hopeful immunomodulatory effect towards repairing cartilage and restoring joint function. Additionally, indomethacin (IMC), a nonsteroidal compound, has been considered as a potent therapeutic agent that exhibits significant antipyretic properties and analgesic effects. The target of the current research is to assess the antiarthritic efficacy of BM-MSCs (10^6^ cells/rat at 1, 6, 12 and 18 days) and IMC (2 mg/kg body weight/day for 3 weeks) either alone or concurrently administered against complete Freund's adjuvant-induced arthritic rats. Changes in paw volume, body weight, gross lesions, and antioxidant defense system, as well as oxidative stress, were assessed. The Th1 cytokine (IL-1*β*) serum level and Th2 cytokine (IL-4) and Nrf-2 ankle joint expression were detected. In comparison to normal rats, it was found that the CFA-induced arthritic rats exhibited significant leukocytosis and increase in paw volume, LPO level, RF, and IL-1*β* serum levels. In parallel, arthritic rats that received BM-MSCs and/or IMC efficiently exhibited decrease in paw edema, leukocytosis, and enhancement in the antioxidant enzymatic levels of SOD, GPx, GST, and GSH in serum besides upregulation of Nrf-2 and anti-inflammatory IL-4 expression levels in the ankle articular joint. Likewise, these analyses were more evidenced by the histopathological sections and histological score. The data also revealed that the combined administration of BM-MSC and IMC was more potent in suppressing inflammation and enhancing the anti-inflammatory pathway than each agent alone. Thus, it can be concluded that the combined therapy with BM-MSC and IMC may be used as a promising therapeutic choice after assessing their efficacy and safety in human beings with RA, and the antiarthritic effects may be mediated via modulatory effects on Th1/Th2 cytokines, ozidative stress, and Nrf-2.

## 1. Introduction

Rheumatoid arthritis (RA) is a predominant inflammatory disorder that is accompanied by a relapsing and remitting course of joint inflammation and synovitis, swelling, autoantibody production, bone dysfunction, and cartilage degradation [1]. RA is accompanied with systemic and additional extraarticular complications of some organs including the lungs and heart. Such systemic manifestations reduce the quality of life and are responsible for disability, early mortality, and socioeconomic costs [2]. The prevalence of RA is more common in the aged people; around 1.3 million adults are suffering from RA as reported by the National Health Institutes [3]. Probably, more than 1% around the world has been established with RA [4]. Up till now, the pathogenesis of RA is unknown; several etiological causes have been involved such as genetic, as well as environmental, factors, infectious agents, heat shock proteins, and sex hormones. Nonetheless, such causes, by themselves, are insufficient to explain the etiology [5]. Among other models, arthritis induced in rodents via injection with Complete Freund's adjuvant (CFA), shares the majority of disease similarities with that of human; thus, this makes it the most appropriate model for inducing arthritis, and it is recommended for studying and testing various therapeutic agents against RA [6–8]. Currently, there is no optimal therapy for RA except for systemic immune suppressants. The most widely used medications such as nonsteroidal anti-inflammatory drugs (NSAIDS) besides the biological agents (e.g., antitumor necrosis-*α* antibody) are helpful in relieving the symptoms, but serious adverse reactions are associated with extended use of these medications. Furthermore, these drugs are costly, and not all patients are reacting well to them [9].

Because of these restrictions, the need for other treatment modalities has emerged in an attempt to develop a treating agent that is as effective as those conventional drugs but devoid of their serious side effects. One of these recently tried approaches is the use of mesenchymal stem cells (MSCs). Bone marrow-derived MSCs (BM-MSCs) are nonhematopoietic multipotential progenitor cells that have positive effects on the reconstruction and integration of the host tissue. They are capable of differentiating into various mesenchymal tissues, including osteocytes, chondrocytes, and adipocytes, thus repairing the cartilage and bone simultaneously [10]. Also, BM-MSCs modulate immune cell responses such as natural killers (NK), antigen presenting cells, T lymphocytes, and B lymphocytes [11, 12]. BM-MSCs, thus, were of considerable importance for both the diagnosis and recognition of the inflammatory autoimmune diseases as well. In osteoarthritis, for example, normal BM-MSCs can help restore damaged cartilage and minimize the low-grade-related synovial inflammation recently verified by a human clinical trial [13]. Such advantages introduced them as a novel therapeutic choice and a promising tool for the prolonged RA treatment.

Moreover, indomethacin (IMC), l-(p-chlorobenzoyl)-5-methoxy-2 methylindol-3-acetic acid), is one of the NSAIDs that were extensively used in both inflammation and pain management. It was introduced in 1963 as a potent agent in treatment of many inflammatory disorders including osteoarthritis, ankylosing spondylitis, gout, acute musculoskeletal disorders, degenerative joint diseases, and RA. IMC has prominent analgesic, anti-inflammatory, and antipyretic properties via inhibiting the activity of cyclooxygenase and, thereby, inhibiting the synthesis of inflammatory mediators [14]. However, therapeutic effects of IMC in reducing inflammation and edema are brilliant, whereas the extended use of these NSAIDs may result in serious side effects, including gastrointestinal harms and cardiovascular toxicity. Thus, configuring new dosing approaches and reducing the side effects of NSAIDs became of growing importance [15]. Therefore, in our study, we administered IMC at a lower dose (0.2 mg/kg body weight (b.w.)) day after day to adjust and control this limitation.

In conductance with the previous literature, the present study aimed to assess the combinatory antiarthritic efficacy of BM-MSCs and IMC, in comparison with the supplementation of each alone in CFA-induced arthritis in Wistar rats. The study also tried to scrutinize the roles of IL-1*β* (Th1 cytokine), IL-4 (Th2 cytokine), oxidative stress, antioxidant defense system, and Nrf-2 in the antiarthritic effects.

## 2. Materials and Methods

### 2.1. Animals

The recent experiment was conducted in the period of October 2018 to October 2019 in Egypt, in the Faculty of Science at Beni-Suef University. It included 50 Wistar rats of male gender (120–150 g in weight; 10–12 weeks old). Rats were bought from Egyptian Organization for Biological Products and Vaccines (VACSERA), Helwan Station, Cairo, Egypt. The animals were overseen for two weeks before the starting of the experiment to exclude any intercurrent infection. Rats were accommodated in calibrated cages of polypropylene and kept in controlled circumstances with a humidity around (55 ± 5%); also, room temperature of (22 ± 2°C), and 12-hour lighting and 12-hour darkness cycle. Animals were given drinking water and fed rat chow diet ad libitum. The experimental animals used in the current research were treated in accordance with the Canadian Council's Principals and Guidelines of Animal Care and Experimental Animal Ethics Regional Committee, Faculty of Science, University of Beni-Suef, Egypt, which approved the experimental work. All attempts were made to minimize the number of struggling animals during the study as possible.

### 2.2. Induction of Arthritis

For arthritis induction, animals were inoculated by a subcutaneous injection of 0.1 mL/rat CFA solution (Sigma Chemical Co., St Louis, Mo., USA) into the footpad of the right hind paw as described by Snekhalatha et al. [16] at dose of 1 mg/kg·b.w. For more extensive severity of arthritis, a booster dose (0.1 mL) of emulsion was administered in the second day.

### 2.3. Animal Grouping

The experimental design contained five groups, each consisting of ten rats and described as follow:

  Group 1 (normal): it consists of healthy rats that were given the equivalent volumes carboxy methylcellulose (CMC) daily and orally for 3 weeks and Dulbecco's Modified Eagles Medium (DMEM) intravenously at the 1^st^, 6^th^, 12^th^, and 18^th^ days.
  Group 2 (CFA): it is composed of CFA-induced arthritic rats and was orally given the equivalent volumes CMC daily and orally for 3 weeks and DMEM intravenously at the 1^st^, 6^th^, 12^th^, and 18^th^ days.
  Group 3 (CFA + BM-MSCs): this group consists of CFA-induced arthritic rats that received four doses of BM-MSCs (1 × 10^6^ cells/rat/dose) by intravenous injection through lateral tail vein per rat [17]. Each dose suspended in 0.2 ml DMEM (Dulbecco's modified Eagles medium). Doses were given at the 1^st^, 6^th^, 12^th^, and 18^th^ days after CFA injection.
  Group 4 (CFA + IMC): this group is composed of CFA-induced arthritic rats supplemented orally with IMC daily in a dose of 2 mg/kg body weight (b.w.). IMC was freshly prepared immediately before administration by dissolving in 5 mL of 1% CMC for three weeks. IMC was acquired from Sigma Chemical Company (Sigma Chemical Co., St Louis, Mo., USA).
  Group 5 (AIA + BM-MSCs + IMC): this group consists of CFA-induced arthritic rats that were concurrently supplemented with BM-MSCs and IMC as described in groups 3 and 4.

### 2.4. Isolation and Culture of BM-MSCs

In the current study, our method of isolation, as well culture of BM-MSCs, is based on the procedure of Chaudhary and Rath [18] and our previous publication in 2020 [19].

### 2.5. Assessment of Arthritis Severity (Represented by Paw Edema)

For monitoring of arthritis development, paw volume is measured as a pointer of paw edema and swelling rate. Measurements were taken at various times at day 7, 14, and 21 following arthritis induction by CFA, and day 0 was the first of CFA injection. Hind paw volume was calculated depending on the water replacement method by subtracting the difference in water volume before and after applying the paw in a calibrated cylinder containing specific volume of isotonic saline. Edema or swelling for the CFA rats was compared to the nonarthritic normal group, while the arthritic-treated rats were compared to the CFA group. The rats were anesthetized by ether inhalation before measurement.

### 2.6. Histopathological Investigation

At the end of the study, at day 21 after induction of arthritis, rats were sacrificed and right hind leg ankle joints of 4 rats from each group were detached and placed for 48 hours in formalin 10% buffered. Decalcification of bone was performed using 10% formic acid. This solution was changed two times per week and for two weeks, and the end of the decalcification process was physically considered using a surgical blade. When decalcification completed, the specimens washed away with PBS, then dehydrated with a graded ethanol series, and embedded in wax cubes made of paraffin. Finally, 5 *µ*m thicknesses sagittal sections were prepared and then stained with haematoxylin and eosin (H & E).

Histological checkups were blindly performed by a pathologist including synovial inflammation, cartilage, and bone damages. Sections were graded in accordance with the system described by Sancho et al. [20] for inflammation (mononuclear cell infiltration), synovial hypertrophy (pannus formation), and erosion of bone, as well as destruction of cartilage. A scale of 0–3-point was used for each parameter (0 grade was considered normal, grade 1 was considered mild inflammation, grade 2 was moderate inflammation, and grade 3 for severe inflammation), and 12 was the maximum possible score.

### 2.7. Detection of IL-4 by RT-PCR

The mRNA expression level of IL-4 in relation to the housekeeping gene *β*-actin (*β*-actin) was determined using semiquantitative Reverse transcription-polymerase chain reaction (RT-PCR).

#### 2.7.1. Ribonucleic Acid (RNA) Isolation

RNA was extracted totally from 3 ankle joints of three different rats in each group using the Thermo Scientific GeneJET RNA extraction kit purchased from Thermos Fisher Scientific Inc., Rochester, New York, USA, according to the manufacturer's instructions. In liquid nitrogen, samples were homogenized and then lysed in lysis buffer solution that contains guanidine thiocyanate, a chaotropic salt which protects RNA from endogenous RNases. The obtained lysate was then mixed with ethyl alcohol and loaded on a purification column. Both the chaotropic salt and the ethyl alcohol made RNA bind to the silica membrane as the lysate is spun through the column. Impurities were subsequently removed away from the membrane by washing the column with washing buffer solution. Then, pure RNA was eluted under low ionic strength conditions with nuclease-free water. Also, the amount of purified RNA was quantified by using a UV Spectrophotometer according to the following formula: RNA *μ*g/*μ*l = O.D. 260 nm *X* (40 *μ*g RNA/ml) × dilution factor/1000. To ensure the high purity of the isolated RNA, we checked the purity of RNA and should be ranged between 1.8 and 2.0. By the end, 0.5 *μ*g of purified RNA was used for production of complementary deoxyribonucleic acid (cDNA) kept at −20°C, for further assay of the mRNA.

#### 2.7.2. Reverse Transcription-Polymerase Chain Reaction (RT-PCR) Analysis

cDNA was prepared by reverse transcription and amplified by using the Thermo Scientific Verso 1-Step RT-PCR Reddy Mix kit obtained from Thermo Scientific Inc, Rochester, New York, USA. The final reaction volume was 50 *μ*l. The mixture of reaction consisted of 2*X* 1-Step PCR Reddy Mix (25 *μ*l), Verso Enzyme Mix (1 *μ*l), RT Enhancer (2.5 *μ*l), sense primer (10 *μ*M) (1 *μ*l), anti-sense primer (10 *μ*M) (1 *μ*l), nuclease-free water (17.5 *μ*l), and template RNA (2 *μ*l). The PCR tubes of the reaction were located in a double-heated led thermal cycler, and a sequence of reactions occurred including verso inactivation at 95°C for 2 min followed by 35 cycles initial denaturation at 95°C for 20 sec, then at 50–60°C for 30 sec, and at 72°C for 1 min, finally, followed by 1 cycle at 72°C for 5 min. After that, we separated the PCR products by electrophoresis on agarose gel (1.5%), and the cDNA bands were observed using a UV transilluminator in a dark chamber. Gel images were investigated and analyzed by scanning densitometry (Gel Doc. Advanced ver 3.0). The primer sequences for IL-4 mRNA are as follows: forward: 5′GGAACACCACGGAGAACG3′ and reverse: 5′GCACGGAGGTACATCACG3′. The primer sequences for *β*-actin mRNA are as follows: forward: 5′TCACCCTGAAGTACCCCATGGAG3′ and reverse: 5′TTGGCCTTGGGGTTCAGGGGG3′. All the primers used in this experiment were synthesized by Sangon Biotech (Shanghai, China) [21].

### 2.8. Hematological Parameters and Cytokine Analysis in Blood

By the end of experiment (after 21 days), the rats were anesthetized under mild diethyl ether and blood was obtained from the jugular vein. Part of the blood was collected in tubes containing ethylenediamine tetraacetic acid solution (EDTA) (50 *µ*L of 15% EDTA 2.5 mL blood) for total and differential leukocyte counting. Total count of leukocyte (TLC) was calculated through a gentian solution diluted sample loaded on a Neubauer hemaocytometer slide for counting, while differential count of leukocyte (DLC) was prepared using Giemsa stain [9].

Although, the other remaining amount of blood was collected directly in pipes without anticoagulant and centrifuged for 15 minutes at 3000 rpm. The clear nonhemolyzed supernatant or sera of different samples were quickly removed and maintained at −30°C. Determination of serum RF and 1L-1*β* levels was performed in normal and arthritic control, as well as the arthritic-treated rats, using specific enzyme-linked immunosorbent assay (ELISA). The kits were purchased from R & D Systems (R & D Systems, Inc., Minneapolis, MN, USA) in accordance with standard procedures.

### 2.9. Oxidative Stress and Antioxidant System

The malondialdehyde (MDA) level was used as an indicator of the lipid peroxides (LPO) level in tissues [22]. Also, the glutathione (GSH) content in serum was determined according to the method of Beutler et al. [23]. Moreover, the antioxidant enzymes including glutathione peroxidase (GPx), superoxide dismutase (SOD), and glutathione-S-transferase (GST) activities in sera were assessed based on the methods reported by previous publications [24–26].

### 2.10. Western Blot Analysis

The western blot technique was used to determine the amount of Nrf-2 protein. In short, proteins were extracted from 3 ankle joints of the right hind leg of three rats in each group using ice-cold radioimmunoprecipitation assay (RIPA) buffer (Beyotime Biotechnology, China) containing 0.1 percent phenylmethylsulfonyl fluoride. Equivalent protein amounts (30 *μ*g) were divided using 10% sodium dodecyl sulfate polyacrylamide (SDS-PAGE) gel electrophoresis. Next, the proteins loaded on the gel were shifted onto membranes of polyvinylidene fluoride (PVDF). Then, overnight, the membrane was probed at 4°C with the Nrf-2-specific primary antibody (cat. no. 68817; Thermo Fisher Scientific). After washing with tris-buffered saline with Tween 20 (TBST) for three times, the blots were prepared for incubation with horseradish peroxidase-conjugated secondary antibodies (1:5000, Santa Cruz Biotechnology, CA) at room temperature 25°C for 30 minutes. The blots were washed again, and then, the signal of the chemiluminescence was visualized with an X-ray film [27].

### 2.11. Statistical Analysis

Statistical research was carried out with the package of statistics for social science version 22 (SPSS, Chicago, IL) statistical program. Comparisons among the mean of different groups were carried out according to one-way analysis of variance (ANOVA) followed by Tukey's postmultiple comparison test. Values are expressed as the mean ± standard error of mean (mean ± SE). *P* < 0.05 was regarded a statistically significant result.

## 3. Results

### 3.1. Treatments Effect on Paw Volume

**Figure 1:**
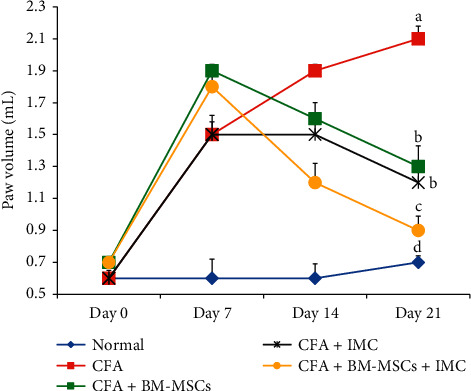
Effect of BM-MSCs and/or IMC on right hind paw volume in CFA-induced arthritic rats. Means, which have different symbols, are significantly different at *P* < 0.05.

Rats of all arthritic groups developed arthritis after CFA induction. The CFA-induced arthritic rats exhibited significant (*P* < 0.05) increase in paw volume (edema) maintained for 21 days when compared with the normal group (Figure 1). In contrary, by the end of the experiment, the BM-MSCs- and/or IMC-treated arthritic rats distinctly reduced the paw edema with inhibition percentages of 36.97, 40.20, and 57.98 %, respectively, in comparison with the CFA-induced arthritic group (Figure 1).

### 3.2. Effect of Treatments on Gross Lesions of the Paw and Ankle Joint

**Figure 2:**
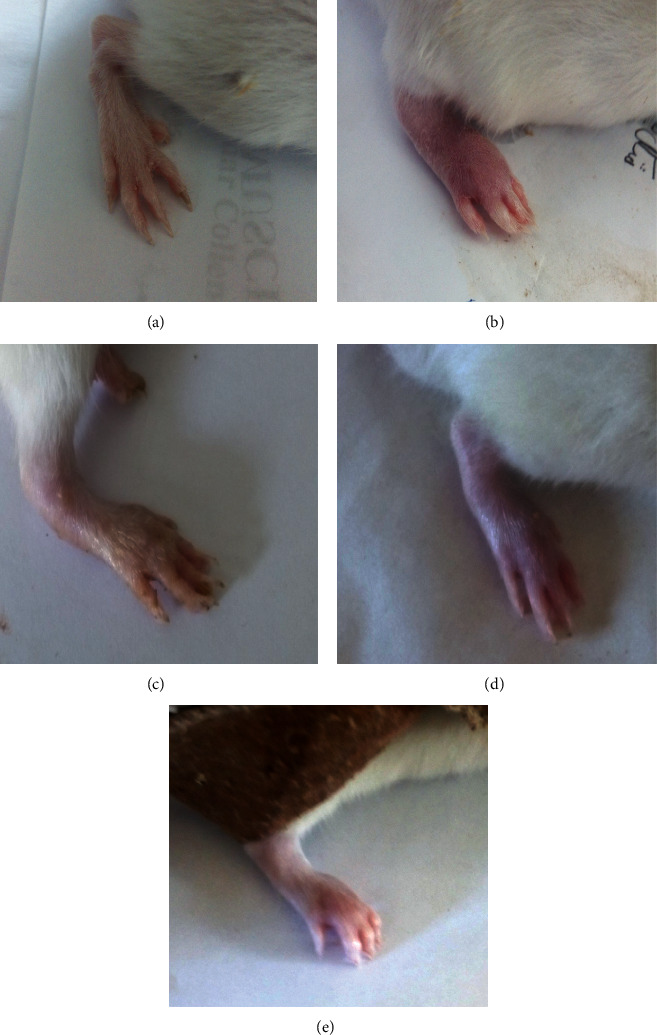
Effect of BM-MSCs and/or IMC on gross lesions in CFA-induced rats showing (a) normal rat, (b) CFA-induced arthritic rat, (c) CFA + BM-MSCs-treated rat, (d) CFA + IMC-treated rat, and (e) CFA + BM-MSCs + IMC-treated rat.

Edema and redness of the right hind paw and ankle joint acted as external measures for determining the severity of the arthritic inflammatory model. The CFA-induced arthritic control group displayed gradual decrease in both of them following BM-MSCs and/or IMC treatments (Figure 2).

### 3.3. Histopathological Changes and Arthritic Score

#### 3.3.1. Histopathological Changes

**Figure 3:**
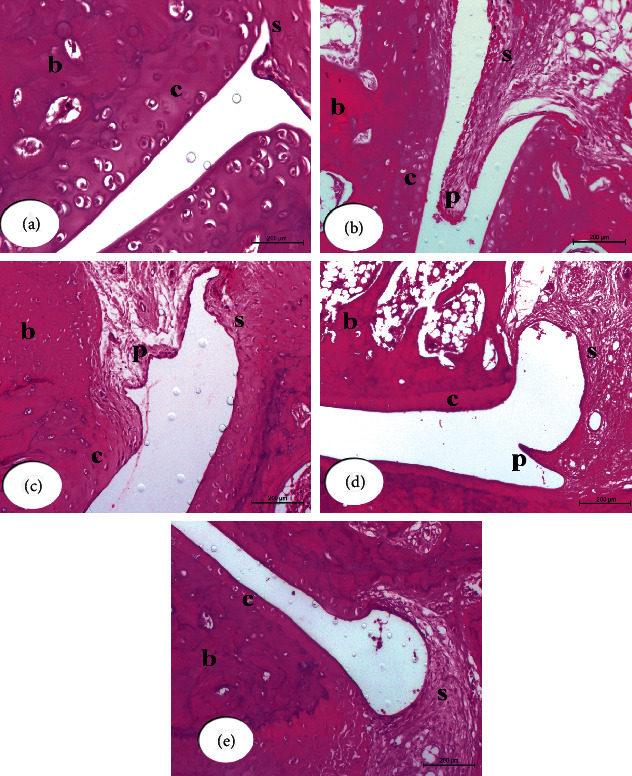
Photomicrographs of (H & E 200×) stained sections of right hind leg ankle joints show histopathological effects of BM-MSCs and/or IMC treatments on CFA-induced arthritic rats. Ankle joint section from normal rat (a) shows normal structure of the synovial membrane (s), cartilage (c), and bone (b). Ankle joint section from CFA-induced arthritic rat (b) shows the substantially expanding synovial membrane with sever pannus formation (p). Ankle joint section from CFA + BM-MSCs rat (c) shows the synovial membrane with mild infiltration and pannus formation (p). Ankle joint section from CFA + IMC rat (d) shows moderate infiltration and pannus formation. Ankle joint section from CFA + BM-MSCs plus IMC rat (e) shows nearly normal histological structure of the hind ankle joint with a normal synovial membrane and mild cellular infiltration.

Hematoxylin and eosin (H & E) stained sections of the right hind leg ankle joint from normal control rats showed no inflammation. In contrast, the CFA-induced arthritic rats exhibited striking histopathological alterations in the form of hyperplasia of the synovium with infiltration of a large number of inflammatory cells, severe formation of pannus, and widespread cartilage destruction. On the other side, the CFA-induced arthritic rats treated with IMC showed less severe pathological arthritis with moderate inflammation, while both rats treated with BM-MSCs and those in concurrently administered group (BM-MSCS plus IMC) showed mild degrees of arthritis (Figure 3).

Microscopically, CFA-induced arthritic rats revealed synovitis characterized by proliferation of the synovial membrane, arranged in 2 to 3 cell lining layers besides proliferation of the underneath vessels of blood that linked with perivascular edema and diffused infiltration of mononuclear cells. In many samples, the exudates of the inflammatory cells spread to involve entire periarticular soft tissues of the connective tissue and muscles. There was detachment in some regions of the synovial membrane and mild proliferative lesions of fibroblast-like cells. Also, the pannus development was in the form of one or more proliferating granulation tissues containing inflammatory cells and hyperplastic synovitis at the joint-cartilage boundary and at the cartilage-bone level. Many arthritic rats' articular cartilage had an irregular articular surface and displayed superficial fibrillation followed by cell death or proliferation, which, in some cases, spread to the articular cartilage's mildly formed part. In addition, osteoclast activity and fibroplasia visualized the joint bone injury. In contrast, arthritic rats treated with BM-MSCs, as well as the concurrently administered group BM-MSCS plus IMC, showed the aforementioned histopathological arthritis lesions but with mild degrees, while arthritic rats treated with IMC showed moderate degrees of those lesions (Figure 3).

#### 3.3.2. Histopathologic Score of Arthritis

**Figure 4:**
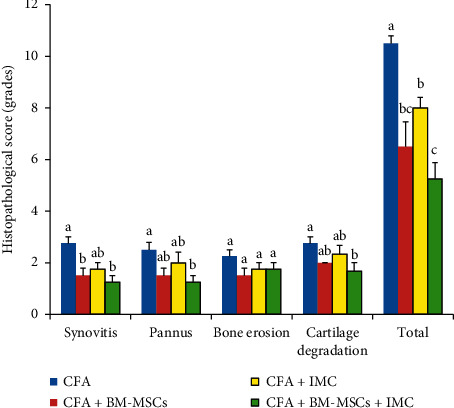
Effect of BM-MSCs and/or IMC on the arthritic score in CFA-induced rats.

Histopathologic analysis comprising synovitis (synovial hyperplasia), pannus formation, cartilage destruction, and bone erosion were scored by a blinded observer on a 0–3 scale in H & E-stained sections. The present data revealed that the arthritic-treated rats significantly exhibited decrease in the total histopathological score as compared to those in arthritic control; the treatment with BM-MSCs + IMC was the most potent (Figure 4). In detail, all cures obviously lessened the synovitis, decreased pannus formation, and reduced cartilage degradation compared to CFA rats. However, bone erosion in treated rats displayed a nonsignificant change (*P* > 0.05) compared with the CFA animal group (Figure 4).

For each score, means, which have different symbols, are significantly different at *P* < 0.05.

### 3.4. Assessment of Total and Differential Leukocyte (TLC and DLC) Count

**Figure 5:**
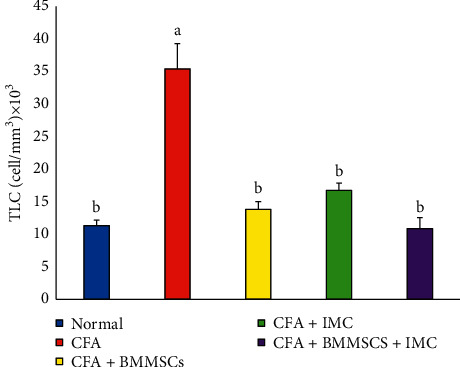
Effect of BM-MSCs and/or IMC on TLC count in CFA-induced rats.

TLC was significantly (*P* < 0.05) increased in the CFA-induced arthritic group with a change percentage of 212.07% when compared to those of the normal control group. However, the CFA-induced arthritic rats supplemented with BM-MSCs and/or IMC showed a marked decline of leukocytosis to close normal levels, and the BM-MSCs + IMC-treated group was the most potent to overcome the elevation of leukocyte total count (Figure 5). Furthermore, differentiation of leukocytes showed a noticeable rise in lymphocyte, as well as neutrophil, count in the CFA-rats two times more than normal rats. However, the BM-MSCs either alone or BM-MSCs plus IMC to arthritic rats significantly improved all leukocytes' counts (lymphocyte, as well as neutrophil and others) near to normal ranges, while IMC supplementation alone declined lymphocyte, neutrophil, and eosinophil count clearly and did not significantly affect monocyte and basophile counts in comparison with the CFA-indued arthritic group (Table 1). The treatment with BM-MSCs + IMC was the most effective in decreasing the elevated lymphocyte and neutrophil counts.

Means, which have different symbols, are significantly different at *P* < 0.05.

### 3.5. Effect on LPO and Antioxidant Status

Table 2 shows the effect of BM-MSCs and/or IMC treatments on oxidative stress and antioxidant defense system markers in serum samples of CFA-induced arthritic rats.

The MDA level showed a ten-fold increase (989.52%) in CFA-induced rats as compared with the normal rats. On the other hand, the treatment of arthritic rats with BM-MSCs and/or IMC treatments decreased (*P* < 0.05) the elevated LPO level significantly in all treated groups with change percentages of −72.99%, −61.06%, and −46.37% for BM-MSCs plus IMC-, BM-MSCs alone-, and IMC alone-treated groups, respectively. Hence, BM-MSCs plus IMC followed by BM-MSCs seemed to be more efficient in improving the LPO level in arthritic animals.

The administration of CFA to Wistar rats significantly (*P* < 0.01) decreased the serum GSH content (−19.75%). While the treatment of CFA-induced arthritic rats with IMC did not significantly (*P* > 0.05) affect the GSH level (6.18%), the treatment with BM-MSCs alone or in combination with IMC produced a significant (*P* < 0.05) increase in the lowered GSH level. The combinatory effect of BM-MSCs was the most potent in increasing the GSH level.

The activities of antioxidant enzymes including SOD, GPx, and GST activities exhibited significant decreases in CFA-induced arthritic rats; the recorded percentage changes were −54.19%, −58%, and −34.71%, respectively, as compared to normal animals.

SOD activity significantly increased (*P* < 0.01) in CFA-induced arthritic rats treated with BM-MSCs + IMC (112.68%), while it was not significantly (*P* > 0.05) decreased as a result of treatments with either BM-MSCs (49.30%) or IMC (64.79%).

GPx activity exhibited significant increase (*P* < 0.01) in CFA-induced arthritic rats treated with BM-MSCs, IMC, and BM-MSCs + IMC recording percentage decreases of 69.00, 66.00, and 121.00%, respectively, as compared with CFA-induced arthritic control.

The lowered GST activity in serum of CFA-induced arthritic rats showed an increase as a result of treatment with BM-MSCs, IMC, and BM-MSCs + IMC. But, the effect was significant only due to treatment with the combination of BM-MSCs and IMC.

Overall, the BM-MSCs + IMC was the most potent in suppressing the LPO level and enhancing the antioxidant defense system.

### 3.6. Effect of Treatments on IL-1*β* and RF Expression Levels

**Figure 6:**
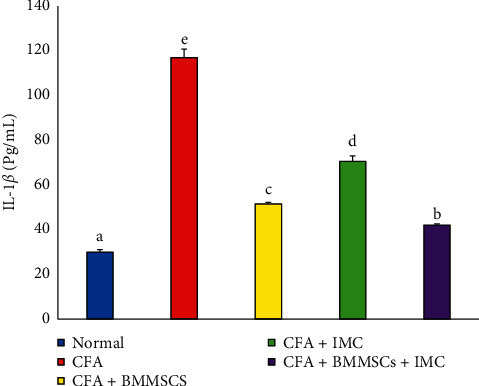
Effect of BM-MSCs and/or IMC on the IL-1*β* level in CFA-induced animals. Means, which have different symbols, are significantly different at *P* < 0.05.

**Figure 7:**
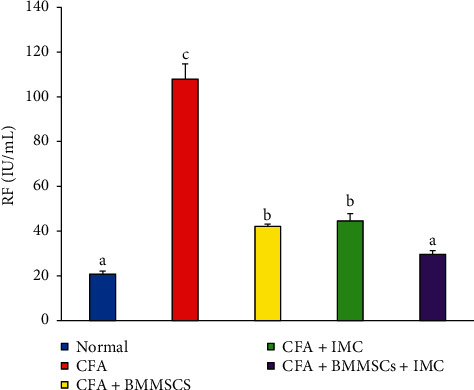
Effect of BM-MSCs and/or IMC on the RF level (mIU/mL) in CFA-induced animals. Means, which have different symbols, are significantly different at *P* < 0.05.

IL-1*β* and RF concentrations in serum were estimated using a standard ELISA and represented as in Figures 6 and 7. CFA-induced arthritic rats had significantly (*P* < 0.05) elevated IL-1*β* and RF level recording percentage changes of 289.67% and 419.74%, respectively, as compared with those in the normal control group. On the other hand, administrations of MSCs and/or IMC lead to significant reductions in IL-1*β* and RF levels. The concurrent administration was the most efficient than BM-MSC than IMC drug in both IL-1*β* and RF concentration levels.

### 3.7. Evaluation of the IL-4 m-RNA Expression Level and Protein Level of Nrf-2 in Ankle Joints

**Figure 8:**
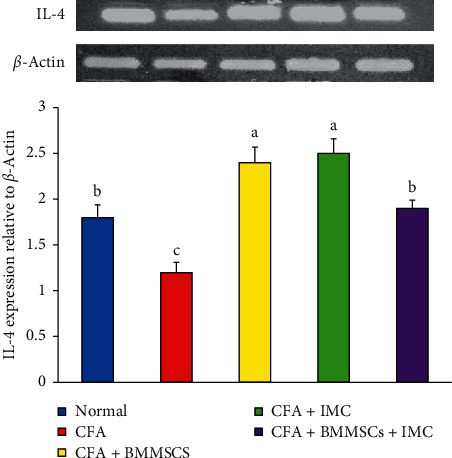
Effect of BM-MSCs and/or IMC on the IL-4 expression level in arthritic rats. Means, which have different symbols, are significantly different at *P* < 0.05.

IL-4 mRNA expression determined in the articular ankle joint by PCR is represented in Figure 8. Data exhibited significant downregulation (*P* < 0.05) of the IL-4 level in CFA animals (−33%) as compared with the normal. Otherwise, IL-4 levels in arthritic rats administered with BM-MSCS plus IMC, BM-MSCs alone, and IMC alone were significantly upregulated recording percentage changes of 58, 100, and 108%, respectively, in comparison with arthritic control rats.

**Figure 9:**
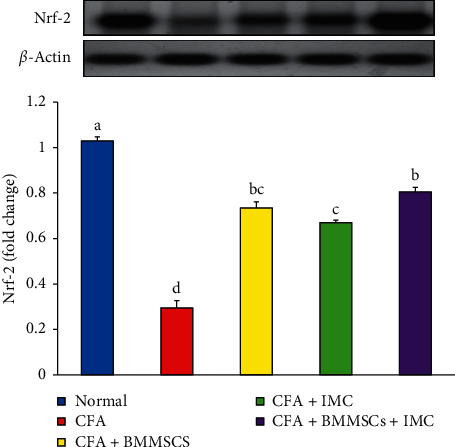
Effect of BM-MSCs and/or IMC on the Nrf-2 expression level in arthritic rats. Means, which have different symbols, are significantly different at *P* < 0.05.

The Nrf-2 protein level in the ankle joint was measured by the western blotting technique that showed marked decline (−63%) in the CFA-induced arthritic rats compared to the normal rats, whereas the supplementation with BM-MSCS plus IMC, BM-MSCs alone, and IMC alone markedly alleviated the Nrf-2 level of these groups with a percentage of increase 249, 127, and 149%, respectively, in comparison with arthritic control rats (Figure 9).

## 4. Discussion

RA is considered an autoimmune inflammatory syndrome causing synovial proliferation, progressive erosion of bone, and destruction of the articular cartilage. The disease affects the quality of life and has a striking impact on societal and economic cost and is considered as an important cause of premature death [28, 29]. Nowadays, treatment with stem cells is considered as one of the most promising signs for the proximate future. This form of therapy could boost or even reverse certain degenerative diseases and probably have applications in alternative and regenerative medicine [19, 30]. MSCs are a remarkable and effective tool in bone and cartilage tissue repair, as they can differentiate into several connective tissues including fat, cartilage, and bone. In addition, they have immunomodulatory and anti-inflammatory effects, self-renewal ability, stemming maintenance, and plasticity for allo- and xenotransplant applications [31]. Also, the use of IMC in the treatment of RA has attracted many researchers because of their analgesic, anti-inflammatory, and antirheumatic influences on the inflammatory disorders. This directed us to evaluate the usefulness of the combination of BM-MSCs plus IMC agents on CFA-induced arthritis as a convenient, easy, and replicable model with short duration of the experiment.

Severe paw swelling (edema), synovial proliferation, accumulation of neutrophils, and bone, as well as cartilage, damage are a marked features of the CFA-induced arthritis model [32]. Here, the right hind paw volume was estimated weekly, and CFA-induced arthritic rats exhibited increased paw volume and swelling in comparison with normal animals. The current data showed that the supplementation of the arthritic rats with BM-MSCs and/or IMC either alone or combined successfully reduced the hind paw volume by the end of the experiment in relation to arthritic rats; thus, the tested agents efficiently suppressed the inflammation inside tissues. Similarly, the research of a previous publication [33] revealed that treatment with IMC decreased the injected paw and contralateral paw edema by 35% and 30%, respectively. Such macroscopic investigation was more elucidated via further microscopic examination of paw sections and their histological score. Histopathologically, rats in the CFA-arthritic control group exhibited severe stage of inflammation described as invasion of leukocytes, synovial hyperplasia, cartilage degradation, and bone resorption. Conversely, animals supplemented by IMC greatly alleviated the joint damage and displayed moderate stage of these lesions. Furthermore, the BM-MSCs-treated arthritic group and BM-MSCs + IMC-treated arthritic group afforded a significant protection against those alterations and exhibited mild stage of such lesions where normal joint space, less infiltration of leukocytes, and an intact appearance of the cartilage were detected. Such observations were more elucidated by the histological score that exhibited a significant reduction in the total score by treatments in this order: BM-MSCs plus IMC > BM-MSCs > IMC as compared to CFA-induced arthritic control rats. Particularly, BM-MSCs plus IMC treatment markedly declined the synovitis, suppressed pannus formation, and reduced degradation of the cartilage as compared to arthritis control rats, while bone resorption was not significantly changed. Hence, such findings obviously evidenced the potency of the tested agents in suppressing the inflammatory reactions inside tissues and illustrated the suppressed inflammatory reactions and paw swelling induced by arthritis.

Likewise, changes in body weight are a valuable index for evaluating disease course and response to an anti-inflammatory drug under research. In the present study, after two weeks of adjuvant injection, the CFA-induced arthritic rats showed observable loss of weight in comparison to normal animals. This finding is in agreement with Da Silva et al. [33] who reported that the adjuvant administered rats developed a significant body weight loss 8 days after injection compared with the healthy control group. This could be attributed to loss of appetite and loss of weight as a result of CFA injection and expansion of arthritis as the study of Da Silva et al. [33] illustrated. On the other hand, supplementation of rats with BM-MSCs and/or IMC treatments either alone or concurrently resulted in remarkable reduction of weight loss in regard with the arthritic group. The BM-MSCs plus IMC was more efficient (44.53%) than IMC (36.97%) and BM-MSCs (34.45%). Our results were entirely consisting with the study of Moases Ghaffary and Abtahi [34] who reported a significant reduction in the average body weight of RA rats when compared with the normal control rats but the extent of weight loss was significantly restricted in RA rats received both caffeine pulsed MSCs and unpulsed MSCs therapies, compared to the vehicle-treated RA animals. Also, Da Silva et al. [33] stated that the weight gain of IMC-treated arthritic rats was higher than untreated arthritic rats but still lower than normal rats. Overall, the macroscopic examinations such as inhibited paw volume and alleviated body weight, as well as the microscopic results, suggested the first indication of the protective and anti-inflammatory action of BM-MSC and/or IMC treatments as therapeutic targets for RA.

In the same line, leukocytes pass to inflammatory sites in response to chemotactic stimuli, causing tissue swelling and oedema, thereby playing a critical role in pathogenesis of both acute and chronic inflammatory disorders [35]. In relation to TLC, as well as DLC, our data revealed that the AIA animals exhibited sever leukocytosis with concomitant increase of lymphocytes, as well as neutrophils comparing to normal animals. These results are in accordance with Pinal et al. [36] who established marked leukocytosis in rats on day 21 after arthritis induction and the study of Franch et al. [37] who explored leukocytosis with higher number and percentage of both neutrophils and lymphocytes in adjuvant-induced rats regarding to normal. Side by side, Pinal et al. [36] informed that leukocytosis and neutrophilia were detected as a result of CFA injection. Such fluctuations in leukocytes are likely due to the inflammatory process present in arthritis involving the fenestration of the microvasculature, element leakage into the interstitial space, and the transfer of white blood cells into inflamed tissues [38]. In this study, our data revealed that these changes were returned near to normal levels in animals treated with test drugs in comparison to the arthritic rats. The BM-MSCs plus IMC and BM-MSCs rats efficiently reversed their alleviated counts to normal ranges whereas the IMC significantly improved neutrophils, lymphocytes, and esinophils but decreased monocytes, as well as basophils, slightly when compared to the normal group. These results recommended the potential anti-inflammatory and anti-arthritic effectiveness against the disease development and progression.

Another factor of RA pathogenesis which cannot be ignored is oxidative stress. Former studies of Holley and Cheeseman [39] and Ali et al. [40] illustrated that accumulation of macrophages and granulocytes in the inflamed region facilitates the formation of reactive oxygen species (ROS) and free radical species (FR), such as superoxide (O^−2^) and hydrogen peroxide radicals (H_2_O_2_). Reactive oxidants are formed in many compartments inside the cell, either normally from internal metabolism or due to external exposure to toxic or pathological insults [41, 42]. In normal conditions, ROS are produced in a restricted way, and some have useful purposes such as acting as essential regulators of both physiological and pathophysiological outcomes [43]. They are developed in response to physiological indications to control processes such as division of the cell, autophagy, stress, immune function response, and inflammation as essential signaling molecules [42]. Inversely, uncontrolled oxidant production results in oxidative stress which also impairs cellular functions and causes cancer, chronic disease, and toxicity development [44, 45]. It is not surprising that the role of oxidative stress or ROS in arthritis serves as a mediator in the pathogenesis of cartilage destruction and tissue damage. A common feature which defines the oxidative stress present in rats is lipid peroxidation or LPO production. MDA is regarded a prooxidant component and used to determine LPO. Generating ROS/RNS is normally stabilized by endogenous antioxidants involving SOD, GSH, GPx, and GST. The first line of antioxidants, GSH and SOD, catalytically scavenge the free radicals (O_2_^−^ and H_2_O_2_). SOD converts the O_2_^−^ to H_2_O_2_ and is widely used during the toxic effects of O_2_^−^ to protect cell damage. Endogenous antioxidant GPx reduced the H_2_O_2_ to form oxidized glutathione and water in the presence of GSH [39]. Imbalance in this process during an exacerbated cell response results in cellular dysfunction and excessive pathological circumstances including bone and cartilage degradation [46, 47]. Furthermore, injection of CFA into rats induces substantial release of ROS and FR at the site of inflammation [48]. Our findings offer good support to these studies because we observed a significant increase in the level of MDA in parallel with marked reduction of antioxidant levels of GSH, GPx, GST, and SOD in serum samples of arthritic control animals in comparison with normal animals. Elevation in MDA levels may be due to extreme accumulation of leukocytes in the blood and inflamed regions that raise LPO production rates and suppress the antioxidant protection system. These observations were also in accordance with our histological analysis showing damage of tissue and intense infiltration of immune cells.

Regarding to the treated groups, the supplementation with BM-MSCs and/or IMC decreased the oxidative damage significantly via suppressing the LPO level in all treated animals. This is in full agreement with the case study of Nejad-Moghaddam et al. [49] who treated the lungs of a subject previously exposed to sulfur mustard gas utilizing MSCs and reported that favorable results were attributed to the antioxidant properties of MSCs, and this was evidenced by reduced LPO levels in the sputum. Moreover, the antioxidant defense mechanism of BM-MSCs and/or IMC therapeutic agents against oxidative stress was achieved mainly via activation of GPx enzyme that played a crucial role in reducing oxidative stress by the elimination of H_2_O_2_ used as a cell death inducing oxidative stimulus. This finding is in consistent with the study of Chang et al. [50] who showed that MSC treatment upregulated the GPx expression in small bowel ischemia/reperfusion (I/R) injury, septic lung injury, sever acute pancreatitis, and Friedreich's ataxia.

Also, Stavely and Nurgali [51] and Kim et al. [52] revealed that MSC can also promote activity of GPx in fibroblasts during oxidative stimuli. Besides, Huang et al. [53] proved that IMC drug was highly effective in enhancing the GPx activity in the AIA animal model. However, the SOD, as well as GST, activities were increased slightly but were not significant in improving the antioxidant processes GPx enzyme. In the current research, it was concluded that BM-MSCs plus IMC was the most potent than either BM-MSCs or IMC in enhancing the antioxidant defense system on the expense of the oxidative stress in tissues and, thereby, inhibiting the subsequent inflammatory processes.

Furthermore, another indicator of the oxidative damage in the body is nuclear erythroid 2-related factor (Nrf-2) protein that was detected in the current study in ankle joint articular tissue. The former data clearly illustrated the pronounced amelioration of the oxidative stress biomarkers, as well as the ability of the tested drugs in scavenging FR and neutralizing ROS. Nrf-2 is a principal regulator of cellular replies against environmental disorders, and it is probable that Nrf-2 activation can protect against factors triggering autoimmune pathogenesis. Activating this signaling pathway, which contributes to detoxification and protective manners, has prompted a wealth of studies on the potential health benefits and therapeutic application of Nrf-2 [54].

Nrf-2 is a key transcription factor that regulates intracellular redox balance. The latter acts as a sensor of oxidative stress mainly present in the cytoplasm; when the level of ROS is elevated, the transcription of antioxidative stress proteins, including SOD^−1^, NQO^−1^, and HO^−^1, is enhanced. These genes play a vital role in prevention of oxidative stress and tissue damage [38, 55]. Nrf-2-targeted genes include genes involved in the synthesis and conjugation of GSH, metabolism of heme and iron, and metabolism and transport of drugs, as well as antioxidant proteins, enzymes, and transcription factors.

**Figure 10:**
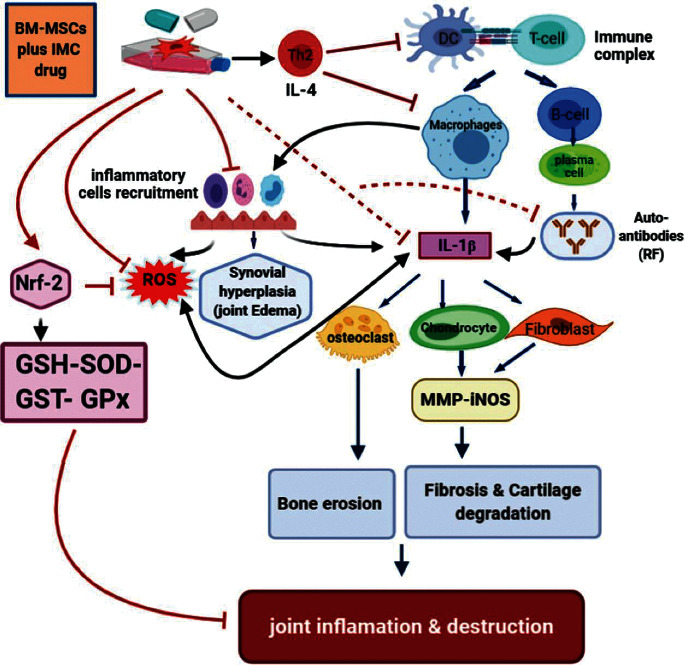
Schematic diagram showing the immunodulatory effect of BM-MSCs plus IMC administered concurrently in CFA-induced arthritis.

Our current research detected a marked downregulation of the Nrf-2 expression level in ankle joint articular tissues of CFA-induced arthritic rats as compared with the normal rats, thus elevating the oxidative damage in this group. On the contrary, the administration of BM-MSCs and IMC either alone or combined together to the arthritic rats produced apparently upregulated expression levels of Nrf-2 in ankle joint tissues as compared to the arthritic control. This finding supports our supposing of the antioxidant effect of the current investigated therapies. In another way, Yoshinaga et al. [56] stated that IMC may be an appropriate therapeutic strategy for choroidal neovascularization (CNV) because of its antiangiogenic effect and that the Nrf-2 signaling is a contributing underlying mechanism. In addition, Wang et al. [57] found that BM-MSCs transplantation in Spinal Cord Injury (SCI) in rats significantly increased the expression of Nrf-2 protein, thereby inhibiting oxidative stress triggered by SCI and promoting spinal cord repair. Overall, these results validate our choice of using BM-MSCs or IMC either alone or combined in inflammatory conditions as reference drugs via their ability of upregulating the Nrf-2 pathway and, therefore, suppressing the oxidative stresses occurred as a result of the disease development (Figure 10).

Furthermore, RA is an autoimmune illness that is well known with formation autoantibodies in serum, as well synovial fluid (SF), samples in 50–80% of RA patients. RF was the first described autoantibody system in RA and focused on Fc portion of human IgG. Despite its suboptimal specificity, the RF occurrence was considered so representative for RA and included in the criteria of the ACR classification in 1987 [58]. Then, it has been included in the 2010 American College for Rheumatology European League Against Rheumatism (ACREULA) classification criteria. However, the RF has been studied for decades, and its role in the pathogenesis of RA remains incompletely known. RF is likely to exert its pathogenic properties via the creation of immune complexes. Then, proinflammatory cytokines, such as TNF-*α* and IL-1*β*, are stimulated and contributing in chronic inflammation and bone destruction [59].

Additionally, cytokines play a crucial role in the pathogenesis of the disease. RA is well known for the ongoing influx of immune cell (monocytes and lymphocytes) into the joints [60]. The process of inflammation is tightly controlled frequently, utilizing both mediators that induce and sustain inflammation which called inflammatory cytokines (TNF-*α*, IL-1*β*, and IL-6) and mediators that shut down the process and called anti-inflammatory cytokines (IL-10 and IL-4). In chronic states of inflammation, an inequality between the two mediators resulting in cellular damage, cartilage and bone destruction, and inflammation [61].

IL-1*β*, the most significant cytokine in pathogenic arthritis progression, has been reported to be correlated with disease behavior such as morning stiffness period [62]. This Th1 cytokine is mainly secreted by macrophages and plays a dominant role in infiltration of inflammatory cells, as well as destruction of bones and cartilage. It also stimulates nuclear factor-B ligand receptor activator on macrophages to differentiate into osteoclasts which resorb and damage the bone. In addition, IL-1*β* prompts the production of inducible nitric oxide (iNOS), prostaglandins (PGE-2), and matrix metalloproteinase (MMP), thus inducing the degradation of the cartilage (Figure 10) [63, 64]. In comparison, IL-4 is an anti-inflammatory cytokine produced by Th2 helper and reduces IL-1 and TNF-*α* production and inhibits cartilage damage. Similarly, the data reported by Chomarat et al. [65] suggested that IL-4 inhibited the development of IL-1 and increased the expression of its receptor antagonism, and both actions would decrease inflammation in synovial culture samples from patients with RA. Hence, IL-4 could be considered as a potential tool for treating autoimmune diseases as evidenced by its protective effect in murine models of RA [66, 67].

In harmony with all our preceding results, data of the arthritic control group showed a significant elevation in the RF level detected in serum in parallel with a remarkable upregulation of IL-1*β* and downregulation of the IL-4 protein level expressed in joint articular tissues as compared to the normal one. These findings are a strong indicator of prominence of the inflammatory pathway on the expense of the anti-inflammatory one as a result of CFA administration. However, the rats supplemented with BM-MSC and IMC either alone or concurrently exhibited a noticeable shift toward the anti-inflammatory pathway and predominance of Th2 on Th1 as evidenced by the apparent upregulated IL-4 and downregulated IL-1*β* expression level in joints accompanied with a significant decline of the RF level in sera of the treated rats compared to the CFA arthritic rats. In accordance with our study, Hoogduijn et al. [68] added that MSCs can alter the cytokine secretion profile of immune cells such as raising the secretion of suppressive cytokines (IL-4 and IL-10) and decreasing the secretion of TNF-*α* and interferon-*γ* (IFN-*γ*) secretion. Besides, it was reported by Selleri et al. [69] and Zhang et al. [70] that the coculture of macrophages with MSCs induces production of M2 macrophages, which upregulates the phagocytic activity and secretion of Th2 cytokines and downregulates levels of Th1 cytokines, such as IL-1*β*, IFN-*γ*, TNF-*α*, and IL-12. Such modulation of the immune response suggests their enhancement of Th2 signaling which directly inhibits the Th1 cascade reaction via suppressing the inflammation propagation (Figure 10). Nrf-2, which has enhanced suppression by the effect of BM-MSCs and IMC, is able to suppress oxidative stress and inflammation and activate the antioxidant defense system (Figure 10). These results represented and introduced a clear interpretation of the efficiency of BM-MSC and/or IMC as antiarthritic and anti-inflammatory agents in overcoming the course of RA.

## 5. Conclusions

Taken together, the present study evidenced that the concomitant treatment with BM-MSCs and IMC administration is more effective in producing anti-inflammatory and antiarthritic effects other than use of each alone during the course of RA in CFA-induced arthritic Wistar rats. This suggestion was supported through the obvious inhibition of the inflammatory signs such as hind paw swelling, weight loss, oxidative stress (LPO), and inflammatory cytokines (RF and IL-1*β*). Moreover, this tested combination succeeds to monitor the disease progression through alleviation of the antioxidant enzyme levels (GPx, SOD, GSH, and GST) and upregulation of Th2 anti-inflammatory cytokine (IL4) and Nrf-2 in joint articular tissues as well. Likewise, the combined treatment alleviated the synovitis, pannus formation, and cartilage destruction occurred as a result of the disease. Eventually, BM-MSCs concurrently administered with IMC could provide an additional promising therapeutic strategy for RA. However, further research should be performed to clarify the mechanisms of the antirheumatic activity of BM-MSCs plus IMC specifically to overcome RA.

## Figures and Tables

**Table 1:** Effect BM-MSCs and/or IMC on DLC count of CFA-induced arthritic rats.

Leukocyte										
Groups	Lymphocyte	(%) change	Neutrophil	(%) change	Monocyte	(%) change	Eosinophil	(%) change	Basophile	(%) change
Normal	613 ± 46^bc^	–	325 ± 14^b^	–	114 ± 29^ab^	–	68 ± 23^a^	–	15±8^b^	–
CFA	1913 ± 204^a^	2.12	980 ± 97^a^	2.0	153 ± 31^a^	0.35	425 ± 124^b^	5.24	71 ± 20^a^	3.68
CFA + BM-MSCs	761 ± 40^bc^	−0.60	434 ± 51^b^	−0.56	74±5^b^	−0.52	92 ± 18^a^	−0.78	23 ± 12^b^	−0.67
CFA + IMC	838 ± 0.0^b^	−0.56	480 ± 30^b^	−0.51	156 ± 11^a^	0.02	156 ± 30^a^	−0.63	45 ± 11^ab^	−0.37
CFA + BM-MSCs + IMC	445 ± 44^c^	−0.77	431 ± 40^b^	−0.56	83 ± 10^b^	−0.46	98 ± 17^a^	−0.77	29±7^b^	−0.59

(i) Data are expressed as mean ± standard error. Number of samples in each group is eight. (ii) For each type of leucocytes, means, which have different superscript symbols, are significantly different at *P* < 0.05. (iii) Percentage (%) changes were calculated by comparing the arthritic group with normal and arthritic-treated groups with the arthritic group.

**Table 2:** Effect of BM-MSCs and/or IMC on lipid peroxides and antioxidant status of CFA-induced arthritic rats.

Parameters										
Groups	MDA (nmole/L) × 10	% change	GSH (nmole/L) × 10^2^	% change	SOD (U/L) × 10	% change	GPx (U/L)	% change	GST (U/L) × 10^2^	% change
Normal	1.8 ± 0.6^d^	—	43.74 ± 0.94^a^	—	15.5 ± 1.6^a^	—	87.00 ± 3.06^a^	—	9.35 ± 1.19^a^	—
CFA	19.1 ± 3.6^a^	961.11	35.1 ± 1.97^b^	−19.75	7.1 ± 0.9^b^	−54.19	36.17 ± 7.39^c^	−58	6.11 ± 0.42^b^	−34.71
CFA + BM-MSCS	7.4 ± 0.5^bc^	−61.26	42.07 ± 1.17^a^	19.86	10.6 ± 0.7^ab^	49.30	61.00 ± 3.87^b^	69	7.97 ± 0.15^ab^	30.45
CFA + IMC	10.2 ± 1.9^b^	−46.60	37.27 ± 0.96^b^	6.18	11.7 ± 1.2^ab^	64.79	60.00 ± 5.29^b^	66	7.64 ± 0.82^ab^	25.11
CFA + BM-MSCs + IMC	5.2 ± 0.6^cd^	−72.77	43.22 ± 1.75^a^	23.13	15.1 ± 2.5^a^	112.68	80.00 ± 5.51^ab^	121	10.18 ± 1.15^a^	66.74

(i) Data are described as mean ± standard error. Number of samples in each group is eight. (ii) For each parameter, means, which have different superscript symbols, are significantly different at*P*  < 0.05. (iii) Percentage (%) changes were calculated by comparing the CFA-arthritic control group with normal and comparing the arthritic-treated groups with the CFA-arthritic group.

## Data Availability

The data used to support the findings of this study are available from the corresponding author upon reasonable request.
